# The Impact of Dietary Intake and Nutritional Status on the Oral Health of Older Adults Living in Care Homes: A Scoping Review

**DOI:** 10.1111/ger.12821

**Published:** 2025-05-19

**Authors:** Aziza Sallam, Noleen K. McCorry, Michelle Harvey, Anja Heilmann, Caroline Lappin, Clare McEvoy, Gary Mitchell, Sinead Watson, Ciaran O'Neill, George Tsakos, Jayne Woodside, Paul Brocklehurst, Kerry B. D. Campbell, Gerry McKenna

**Affiliations:** ^1^ Queens University Belfast Belfast UK; ^2^ The University of Exeter Exeter UK; ^3^ University College London London UK; ^4^ Department of Health Belfast Northern Ireland UK; ^5^ Bangor University Bangor Wales UK

**Keywords:** care homes, dietary intake, nutritional status, older adults, oral health

## Abstract

**Objectives:**

To review the literature on the relationship between oral health, dietary intake, and nutritional status in older adults in care homes, identifying research gaps.

**Background:**

Many older adults in care homes retain natural teeth but face oral health issues due to challenges in maintaining hygiene and limited dental care access. While the provided diet aims to prevent frailty, it may contain high levels of sugars and fermentable carbohydrates, which can contribute to oral health issues such as dental caries and periodontal disease.

**Methods:**

A scoping review was conducted following PRISMA‐ScR guidelines. MEDLINE, CINAHL, Web of Science, and EMBASE were searched to answer, “What is known about the relationship between dietary intake, nutritional status and oral health in older adults in care homes?.”

**Results:**

Forty‐nine studies (1989–2024) addressed this relationship, with most studies being cross‐sectional (*n* = 43), including two qualitative and one mixed‐method study; six were longitudinal, including one interventional. Findings highlighted the negative impact of oral health problems—xerostomia, dysphagia, and chewing issues—on dietary intake and nutritional status, while interventions like denture provision and professional tooth brushing had positive effects. Longitudinal studies emphasised the harm of sugar intake on oral health, with additional factors like dementia, resident dependency, and staffing issues adversely affecting both oral health and nutrition.

**Conclusions:**

This review highlights the complex relationship between oral health, diet, and nutrition in care homes, emphasising gaps in research on the impact of dietary intake, particularly sugar consumption, on oral health, as well as limitations in study designs.

**Abbreviations**
ADL: Activities of Daily Living
BIA: bioelectrical impedance analysis
BMI: body mass index
CDR: Clinical Dementia Rating
CPS: Cognitive Performance Scale
DFS: decayed and filled primary dental surfaces
DMFT: decayed, missing, filled permanent teeth
FOIS: Functional Oral Intake Scale
GI: gastrointestinal
GOHA: Geriatric Oral Health Assessment
GOHAI: Geriatric Oral Health Assessment Index
HDS‐R: Revised Version of Hasegawa's Dementia Scale
HFA: home for aged
HRQoL: health‐related quality of life
ISE: Index of Social Engagement
LTC: long‐term care
MET: masticatory efficiency test
MDS‐NH: Minimum Data Set for Nursing Home Resident Assessment and Care Screening
MMSE: Mini‐Mental State Examination
MNA: Mini Nutritional Assessment
MNA‐SF: Mini Nutritional Assessment, Short Form
NM Scale: N Geriatric Rating Scale for Mental States
N‐ADL: N Geriatric Rating Scale for Activities of Daily Living
OHAT: Oral Health Assessment Tool
OHIP: Oral Health Impact Profile
OHIP‐14: Oral Health Impact Profile Short Form
ODB: oral disease burden
OHRQoL: oral health‐related quality of life
PCS: problems with chewing and swallowing
PD: periodontal
PEFR: peak expiratory flow rate for pulmonary function
PL: process lead
PDC: professional dental cleaning
QoL: quality of life
VE: videoendoscopic examination

## 1 Introduction

There are over half a million older adults living in care homes across the United Kingdom, many of whom retain some natural teeth, creating a partially dentate population [1, 2]. The oral health of care home residents is significantly worse than that of their community‐living peers, largely due to their reliance on staff for oral care [3]. However, staff may not always provide adequate care due to time constraints, staff shortages, or lack of training [4]. Furthermore, polypharmacy—common among older adults in care homes—can exacerbate oral health issues [5]. Many medications prescribed to older adults have side effects that can contribute to xerostomia, making chewing and swallowing difficult, particularly for those wearing dentures [5]. Additionally, exposed root surfaces and heavy restorative dental treatment are common among care home residents [6]. Also, for individuals living with dementia, cognitive decline poses additional challenges to maintaining good oral health [7].

Added to this, the prevention and management of malnutrition (specifically undernutrition) among care home residents represent significant clinical challenges, given the prevalence of frailty within this population [8]. Undernutrition is defined as a deficit of protein and energy intake, with or without consideration of micronutrient deficiencies [9]. To address frailty and mitigate the risk of undernutrition, care home residents are often provided with diets rich in complex carbohydrates, including refined sugars [10]. These dietary interventions aim to enhance caloric intake and stabilise blood glucose levels, crucial for individuals with frailty who may experience energy fluctuations. However, while these measures may meet immediate nutritional needs, they also pose potential risks, such as dental caries and metabolic disorders, associated with high sugar consumption [11].

In the absence of efficient manual cleaning and decreased protective salivary production, caries on teeth can develop extremely fast [12]. This can cause significant pain and discomfort, leading to the extraction of remaining natural teeth, which in turn has further negative consequences for oral function, including speaking, eating and quality of life [13]. Unfortunately, dental treatment for older adults living in care homes in the UK has been found to be insufficient, with little focus given to prevention [14]. While the National Institute for Health and Care Excellence has made recommendations to help improve the oral health of adults in care homes, there was little mention of the role of dietary intake and nutritional status [15].

Despite these high levels of preventable dental diseases, there has been little critical evaluation of the role of dietary intake and nutritional status on oral health within this population. Therefore, a scoping review was undertaken to assess the scope, breadth, and character of research in this area, with the purpose of identifying, evaluating, and synthesising all relevant information about the link between oral health and nutritional status in older adults living in care homes. To support future research and recommendations, we also looked for gaps in the literature regarding the impact of dietary intake and nutritional status on oral health in this population.

As recommended by the updated Joanna Briggs Institute (JBI) methodological guidance, the PCC (population, concept and context) framework was employed to formulate the following review question [16]: “What is known about the relationship or interaction between dietary intake and nutritional status and oral health of older adults living in care homes?”

We also aimed to answer the following sub‐questions related to older adults in care home settings:

- What aspects of oral health have been examined (in the existing literature) and linked to nutritional status and/or dietary intake?
- Are there other factors that could impact the relationship between oral health and nutrition of older adults in care homes, such as the role of care home staff?
- What do authors recommend to improve the oral health and nutrition of older adults in care homes, or to advance the research area?
- Are there any gaps in knowledge concerning the relationship between nutrition and oral health among older adults in care homes?

## 2 Methods

The scoping review followed Arksey and O'Malley's five‐stage framework, which involves defining the research question, identifying relevant studies, selecting studies based on inclusion criteria, data charting, and reporting the results [17]. The updated JBI methodological guidance has been employed as a reference in identifying the title, review question, and inclusion criteria [16]. The PRISMA extension for scoping reviews checklist was used as a reporting template [16].

The protocol was drafted by the first author (AS) using the JBI guidance and was revised by the second and third authors (NM& GMK).

### 2.1 Eligibility Criteria

Peer reviewed sources were included in the review if they met all of the following criteria:

1. included data on dietary intake and/or nutritional status of older adults in care homes, such as information on types and consistency of food consumed, fluid intake and eating problems.
2. included data on oral health of older adults in care homes such as information based on dental examination, self‐reported oral health problems and dental services provided.
3. involved participants (or participants' data) who were care home residents or health care workers.
4. were primary research studies (including observational, experimental, qualitative or survey based).
5. were published in English.

Studies were excluded if any of the following criteria applied:

- involved older adults in community‐dwelling settings only (living in their own homes).
- included data solely on malnutrition or solely on oral health.
- involved only edentulous older adults (all participants have no natural teeth)
- secondary studies including literature reviews, scoping reviews, or systematic reviews.
- were not published in English.

No specific criteria were applied for age range of participants, only that they were described as ‘older adults’. Studies that involved both dentate and edentate participants were included if there was a separate analysis relating to the dentate group.

### 2.2 Search and Screening

Ovid MEDLINE, CINAHL, Web of Science and EMBASE were searched from 1946–2024. The search was supplemented by scanning reference lists of key review papers.

The searches used the JBI's recommendations to combine the following PCC terms: ‘older adults’ (population), ‘relationship between diet, nutrition and oral health’ (concept), and ‘care homes’ (context). The terms used for the search are shown in the appendix. The search strategy used with databases utilised phrases and keywords, and where appropriate, medical subject headings (MeSH). All primary searches were performed between June–August 2024. The entire search strategy for Ovid MEDLINE, CINAHL, Web of Science, and EMBASE is provided in appendix. Search results were then imported into Covidence software.

Two reviewers (AS& KC) independently screened all titles and abstracts against the eligibility criteria, and disagreements were resolved via discussion with NM. Subsequently, both reviewers independently screened full‐text articles against the eligibility criteria. Disagreements at this stage were resolved through discussion with the third reviewer (NM).

### 2.3 Data Extraction and Presentation

A pilot charting table was created in Excel and adapted through discussion between reviewers in an iterative manner during data extraction.

Data was extracted on study design, study aim, measures used for oral health and nutrition assessment, main findings, and author recommendations (See Table 1). Studies were grouped based on the primary aim of the study. A narrative synthesis based on the Popay et al. framework was compiled to describe the main findings of studies addressing each aim [18]. A narrative summary of oral health and nutrition measures utilised was also compiled.

**TABLE 1:** Data charted for each included source of evidence.

N	Study ID	Title	Location	Design	Participants info	Aim	Measure for oral health	Measure for nutrition	Other measures	Main findings	Author recommendations
Category 1: Studies addressing the impact of oral health on nutrition											
1	Ekelund 1989	Dental state and subjective chewing ability of institutionalised elderly	Care homes in Finland	Cross‐sectional study: Clinical examination and structured interview	480 residents of 24 municipal old peoples' homes in Finland. 153 men and 327 women aged 65–100 years	To investigate the dental state of older adults, and to appraise their chewing ability and inability to eat because of their poor dental health	Dental examination. Denture wearers asked about denture retention and presence of pain. Examination to count FTUs	Interview: questions about whether they eat the food they like (meat, crisps, fruits and vegetables) to check chewing ability		Most of participants have problems with eating crisps and bread due to chewing problems related to denture use and lost teeth	Special attention should be given to the dental condition and masticatory function of older adults in care homes to ensure better physical, psychological and social life
2	Loesche 1995	Xerostomia, xerogenic medications and food avoidances in selected geriatric groups	Michigan, US	Cross‐sectional survey: Clinical examination and structured questionnaire	208 individuals from a VA Dental clinic and 114 from a retirement home 132 from nursing homes, and 75 from an acute care unit Average age was 70 years	To study the relationship between xerostomia, salivary performance, and food avoidance To determine whether xerogenic medications are associated with xerostomia and food avoidance	Questionnaire (oral hygiene, dry mouth and swallowing complaints). Dental examination (counting teeth, DMF, prostheses, implants recorded) Salivary flow measured	Structured questionnaire about eating habits, food and liquid preferences, and food avoidance		Specific medications are associated with xerostomia and/or low salivary flow, and/or avoidance of hard or sticky foods because of chewing difficulties	When assessing the interaction between drugs and salivary glands, it is critical to distinguish between medications that create the sense of dryness but have no impact on the salivary glands and those that might truly impede or change salivary production
3	Mojon 1999	Relationship between oral health and nutrition in very old people	Geneva, Switzerland	Cross‐sectional clinical study: Oral examination, and analysis of existing medical data	324 institutionalised frail older adults (mean age 85 years)	To evaluate the relationship between oral health status and nutritional deficiency	Oral examinations by a dentist to assess mucosa, denture quality, caries and periodontal disease	BMI and serum albumin concentration as markers of malnutrition	Barthel index used to assess physical dependence	Compromised oral function status was associated with nutritional deficiency. Compromised oral function was more frequently found in dependent older adults	A dentist and a dental hygienist should be included in the team of health professionals who care for old people
4	Sheiham 2001	Does the condition of the mouth and teeth affect the ability to eat certain foods, nutrient and dietary intake and nutritional status among older people?	UK	Cross‐sectional survey	753 free‐living and 196 institution subjects aged 65 years and older from randomly selected 178 institutions	To assess how the dental status of older people affected their stated ability to eat common foods, their nutrient intake and some nutrition‐related blood analytes	Dental examination assessing the number, position of teeth and presence of dentures. Interviews by a trained examiner to assess difficulty participants report when eating certain foods	4‐day dietary record of all food and drink consumed). Measurements of the concentrations of certain nutrients in blood and urine. This was applied in both samples		In free‐living older adults, more natural teeth meant better chewing ability and higher nutrient intake. In care homes, food was adapted for those with the most difficulty chewing, levelling nutrient intake across residents	None reported
5	Tosello 2001	Oral functional characteristics and gastrointestinal pathology: an epidemiological approach	France	Cross‐ sectional study	211 from 8 geriatric institutions aged 44–100 years	To study the influence of oral functional performance on the gastrointestinal pathology	Oral examination and participants classified according to denture use and FTUs	Dietary surveys and interviews, to collect detailed information about the quantities of foods and nutrients consumed by the participants over a specific period	GI examination to detect pathologies like reflux, heartburn, and discomfort done by a physician with the assistance of the medical staff	Subjects with natural teeth had fewer gastrointestinal disorders than others. Poorer GI pathology was strongly linked to increased age	Loss of teeth must be compensated by functionally effective dentures to avoid GI problems
6	Kwok 2004	Association between functional dental state and dietary intake of Chinese vegetarian old age home residents	China (Hong Kong)	Cross‐sectional survey	76 older vegetarian Chinese (65‐years old or over) women living at an old age home. Mean age 86.6 ± 5.9 years	To examine the association between dental functional status and dietary intake	Oral examination by a dental surgeon to check DMFT, FTU & chewing difficulties. Subjective masticatory ability assessed through a questionnaire asking participants if they could chew different categories of foods properly	A 24‐h food record captured meals and snacks. Nutrient composition of consumed foods was calculated using food tables	Basic physical characteristics, functional status, and BMI were measured. Subjects were categorised based on dependency levels in activities of daily living	Low functional tooth units FTUs was linked to poor chewing difficulties, eating soft food, and being functionally dependent	Dentures must be made available for and maintained in good working order. An efficient service model would be a mobile dental team to address the accessibility issue
7	Suzuki 2005	Relationship between number of present teeth and nutritional intake in institutionalised elderly	Chiba, and Iwate in Japan	Cross‐sectional study	141 subjects average age is 80 or more (S‐group) in health service facility and patients with mild dementia (SD‐group) in a special nursing home for the older adults	To investigate which factors contributed to their dietary intake, and the relationship between dietary intake and number of present teeth	Oral examination to determine the number of remaining teeth. Participants were categorised based on the number of teeth (fewer than 20 or 20 or more)	24‐h recall captured the types and quantities of foods consumed by residents. The nutritional composition of consumed foods was analysed to determine the intake of essential nutrients		Recovery of chewing ability (by reserving natural teeth and denture use) was essential to maintain nutritional status in S group. In SD‐group, low mood lowers the nutritional status	It is essential to regain the chewing ability in older adults to improve the quality‐of‐life QoL
8	Soini 2006	Oral and nutritional status – Is the MNA a useful tool for dental clinics	Care homes and long‐term care in city hospitals in Helsinki, Finland	Cross‐sectional study	2036 from 92 wards in the private and public nursing homes and 1052 from 53 long‐term care wards. Mean age was 83 years in NH and 81 years in LT	To determine the oral status of residents in nursing homes (NH) and long‐term care wards (LT) and to describe association between oral and nutritional status among them	Oral examination and questionnaire about oral health problems	MNA included information about eating habits and diets. The structure of food eaten was categorised as ‘any food’, ‘soft food’, and ‘pureed or liquid food’		In the population of institutionalised frail older adults, malnutrition was related to both poor oral status and oral health problems	All caregivers who work with older adults should be aware that decreasing functional ability increases the need for assistance in performing daily oral care
9	Dion 2007	Correction of nutrition test errors for more accurate quantification of the link between dental health and malnutrition	France	Observational study	94 residents in 100 care homes in the Alpes Region of France. Age is 60 and older	To quantify the link between tooth deterioration and malnutrition in institutionalised older adults considering errors in MNA	Oral examination, including injuries, diseases, hygiene, number of teeth, dental care needs, and masticatory percentage	MNA	Dependency assessment: using the GIR grid. Disease Status Indicator: Categorised as disease‐free, acute disease, chronic disease, or multiple diseases	After controlling for other risk factors, the risk of malnutrition increased significantly 1.15 times anytime the masticatory % fell by 10 points, which is comparable to losing two molars	Regular dental examination and care should help to maintain oral health and avoid malnutrition
10	Wostmann 2008	Influence of denture improvement on the nutritional status and quality of life of geriatric patients	Two care homes in Germany	Observational study	47 patients (minimum age: 60 years) and with dentures requiring repair or replacement randomly selected from two nursing homes in Germany (19 M and 28 F) Participants have remaining natural teeth	To identify the impact of denture improvement on the nutritional status and OHRQoL in geriatric patients	OHIP‐14 to assess OHRQoL and Masticatory efficiency test MET to evaluate masticatory function	MNA, and serum albumin measured	MMS to screen dementia	MMS remained unchanged, MNA increased slightly after 6 months, Masticatory ability increased. Prosthetic treatment alone will not result in a substantial improvement in the nutritional status of older adults who are dentally compromised	Further studies needed to determine boosting nutritional status of older adults by improved prosthodontic status and dietary consultation
11	Wang 2012	Associations between chewing and swallowing problems and physical and psychosocial health status of long‐term care residents in Taiwan: a pilot study	Long‐term care institutions and public nursing homes in Taipei, Taiwan	Cross‐sectional study	781 residents in community long‐term care institutions and public nursing homes in Taipei, Taiwan. 308 females and 473 males with a mean age of 79 participated	To investigate the status of oral health, nutrition, and quality of life of residents in long‐term care institutions	Interview used by nurses to assess oral hygiene, dental status (e.g., presence of natural teeth, dentures), presence of oral health problems (e.g., oral pain, gum disease)	BMI and mealtime assistance needed	Cognitive Performance Scale (CPS), Index of Social Engagement Activities of Daily Living (ADL) Scale	Problems with chewing and swallowing (PCS) was significantly associated with malnutrition, cognitive performance and social engagement	Future research might focus on education and training to better prepare long‐term care nursing staff to monitor residents' oral health, identify people with PCS, and act as needed
12	Adiatman 2013	Functional tooth units and nutritional status of older people in care homes in Indonesia	4 private care homes in Jakarta, Indonesia	Cross‐sectional study	100 female participants (mean age: 72.4 ± 8.2 years) from 4 private care homes in Jakarta	To investigate the relationship between functional tooth units (FTUs) and nutritional status	Oral examination by a dentist, assessing edentulous state, dental prosthetic status, number of teeth present, decayed or filled and FTUs. Oral hygiene habits assessed through interviews	BMI and MNA. Questionnaire included 18 questions about diet and other nutritional aspects	General health status was evaluated by a registered physician, assessing systemic ailments, medication, and activities of daily living using the Barthel Index	There is a significant relationship between the number of FTUs and nutritional status	Maintaining the posterior occlusion should be emphasised in older adults in order to preserve good nutritional status
13	Saarela 2014	Dentition status, malnutrition and mortality among older service housing residents	Helsinki and Espoo, Finland	Cross‐sectional study with a 3‐year follow‐up	Service housing residents aged 65+ years in the cities of Helsinki and Espoo in Finland (*n* = 2188), 79% were women. The residents´ mean age was 83 years	To assess older service house residents' dentition and its associations with nutritional status and eating habits To explore the prognostic value of dentition status for mortality	Dentition status, oral symptoms, eating habits, and diets were assessed through personal interviews and assessments conducted by trained nurses	MNA: nurses provided information about residents eating habits and diets and BMI calculated	Functional and cognitive status, eating habits, and mobility were collected. Comorbidity was assessed using Charlson's index, and 3‐year mortality data were analysed	Dentition status was significantly associated with the consistency of food offered and with mortality. Group 1 had the highest number of oral symptoms had the least possibilities to oral examinations than G2 & 3	Co‐operation between nursing staff and oral care services is recommended to maintain good nutritional status. Oral examination should be performed to the residents
14	Ogawa 2016	Taste detection ability of elderly nursing home residents	Two different care homes in Osaka, Japan + independently living older adults	Cross‐sectional study	43 older adults (mean age 82 ± 8.5 years) residing in two nursing homes in Osaka and from 949 independently living (mean age 79.9 ± 0.8 years)	To compare taste detection ability between dependently and independently living geriatrics and to evaluate the factors associated with taste sensitivity	Oral examinations by trained dentists, including measurement of maximal occlusal force and assessment of denture usage. Taste sensitivity was assessed using four different tastings	Types of diet were obtained from dietary records conducted by registered dietitian	Medical records and personal interviews provided information on comorbidity and medication	Diseases (including upper respiratory and oral infections, autoimmune diseases, cancer, and Alzheimer's) are related to decreased taste sensitivity in dependent older adults	Understanding taste sensitivity can contribute to improving dietary intake since it affects the appetite of older adults in care homes
15	Huppertz 2017	Association Between Malnutrition and Oral Health in Dutch Nursing Home Residents: Results of the LPZ Study	Germany (Dutch nursing homes)	Cross‐sectional study	3220 older adults' residents from Dutch nursing homes. Mean age 84.3 (±7.4) years, 65–105 years. 70% were women living in psychogeriatric wards	To assess the association between oral health problems and malnutrition in residents of somatic and psychogeriatrics wards	Standardised questionnaire on xerostomia, chewing problems, and artificial teeth problems, was developed by experts	Data on age, BMI, and time‐specific weight loss (%) to determine	Comorbidities and medication were obtained from medical records and personal interviews	Positive associations found between malnutrition and poor oral health especially problems with eating due to (artificial) teeth problems	Future longitudinal research is needed to confirm causation in the relation between malnutrition and oral health
16	Nakagawa 2019	Assessment of Oral Function and Proper Diet Level for Frail Elderly Individuals in Nursing Homes Using Chewing Training Food	Fujita, Japan	Cross‐sectional observation study	100 older individuals aged between 67–96 years, 34 men and 66 women	To investigate the relationship between the ability to press Process Lead (PL) in the oral cavity and the tongue pressure and recommended diet form for older individuals in nursing homes	Process Lead (PL) pressing and chewing tests, maximum tongue pressure (MTP) measurements, and videoendoscopic examination of swallowing (VE)	Nutrition was assessed through oral meal consumption		Participants with inadequate tongue strength and no molar occlusion chewed less and ingested large‐sized particles due to low chewing. Masticatory performance deteriorates when the number of teeth is reduced	The use of PL pressing and chewing tests are useful to determine diet level for long‐term care home residents. However, further studies are needed to validate the assessments
17	Nomura 2019	Consistency of supplied food and dentition status of the elderly in residential care homes	Osaka, Japan	Cross‐sectional study	276 older residents (M = 56; F = 220; mean age, 87.68 ± 5.94 years) from 12 care facilities	Investigation of the relations between dentition status and supplied food consistency among older residents of care facilities	3 dentists conducted oral examinations to determine dentition, use of dentures, and number of tooth or denture contact	The consistency of supplied food was determined based on observations	Care levels determined using standardised criteria outlined in the Japanese Certification of Needed Long‐Term Care	Care levels and number of tooth contact pairs (natural or artificial) were significantly correlated with the consistency of the food supplied to residents. Dentures are necessary for older adults to maintain their health and quality of life	Longitudinal studies are needed to examine interactions among dentition status, swallowing function and supplied food consistency
18	Barbe 2020	Impact of regular professional toothbrushing on oral health, related quality of life, and nutritional and cognitive status in nursing home residents	Germany	Longitudinal study	40 participants 73% F (*n* = 29) mean age of 82 (SD 10) years. At the end of the study, *N* = 34 because of death	To investigate the effectiveness of professional brushing with a professional brush every 3 weeks and determine how it affected older adults' outcomes	Oral examination indicated the number of teeth, prosthetic situation, and periodontal status before and after PDC using three‐headed brush every 3 weeks for 3 months by dental nurses under the supervision of nursing home dentists. OHRQoL assessed using GOHAI	MNA	Cognitive functions assessed using MMSE	Professional brushing by a dental nurse on a regular basis is an effective way to enhance oral hygiene in nursing home residents and may lead to improved nutritional status, GOHAI and quality of life	The favourable effect would be more apparent if the intervals between professional cleanings were reduced while the duration of cleaning increased
19	deMedeiros 2020	Masticatory function in nursing home residents: Correlation with the nutritional status and oral health‐related quality of life	Brazil	Cross‐sectional study	344 older adults from 2 nursing homes in Brazil aged 60 years or more. *n* = 344; mean age 77.7	To evaluate the influence of the presence of teeth and dentures on masticatory function and swallowing in institutionalised older adults	DMFT assessed per WHO criteria. Masticatory performance evaluated with two‐colour chewing gum for 20 cycles. Swallowing threshold determined by peanut chewing cycles. OHRQoL assessed with Brazilian GOHAI and OHIP‐14	MNA‐SF used for nutritional status assessment. BIA used for body composition assessment, including weight, BMI, muscle mass, and body fat percentage		Absence of teeth/dentures negatively influenced masticatory performance and swallowing and reduced nutrient intake and nutritional status of care home residents	The masticatory function recovery through denture rehabilitation is important to improve the well‐being of institutionalised older adults
20	Izumi 2020	Impact of Tongue Pressure and Peak Expiratory Flow Rate on Nutritional Status of Older Residents of Nursing Homes in Japan: A Cross‐Sectional Study	Japan	Cross‐sectional study	52 residents (12 M and 40 F) from 3 care homes in Japan. The mean age 82.2 ± 9.8 years for men and 90.5 ± 5 for women	To investigate the impact of tongue pressure, and pulmonary function on the nutritional status of older adults	Oral examination checked occlusal support, plaque index, and tongue contamination. Swallowing function was tested with the Modified Water Swallow Test (MWST). Maximum tongue pressure was measured using a specialised device	MNA‐SF consists of 6 items: food intake, weight loss, mobility, psychological stress or acute disease, neuropsychological problems, and BMI	Peak expiratory flow rate (PEFR) was measured using a spirometer. ADL and cognitive function were evaluated using the Barthel Index and mini‐mental state examination (MMSE), respectively	Maintaining oral and pulmonary function may be a preventive factor against a decrease in the nutritional status of older frail adults	To identify the precise effects of tongue pressure and PEFR on nutritional status, longitudinal or intervention studies are required
21	Kuijk 2021	Dentition and nutritional status of aged New Zealanders living in aged residential care	New Zealand care homes	Cross‐sectional study	987 participants (65–106 years) represented a national source population of 14,404 individuals	To investigate the association between dentition status and nutritional status in a national survey of older New Zealanders living in aged residential care facilities	Oral examinations by a dentist including assessments for dental caries, periodontal status, and prosthetic use	MNA‐SF	cognitive function assessments, and socio‐economic status measurements were performed	Untreated dental caries was associated with malnutrition. There were no links found between missing teeth and malnutrition. Those who had worse cognitive function or were more dependent on others were more likely to be malnourished or at risk of malnutrition	The length of stay in care homes could be a factor to study, since it could be associated with poor nutritional status
22	Schmalz 2021	Oral Health‐Related Quality of Life, Oral Conditions, and Risk of Malnutrition in Older German People in Need of Care‐A Cross‐Sectional Study	8 nursing homes (including three nursing homes with assisted living) and one mobile nursing service in Germany	Cross‐sectional study	151 participants (age: 84.17 ± 7.8 years)	To assess oral health, nutritional condition, and oral health‐related quality of life (OHRQoL) in older German people in need of care	A single dentist conducted oral examinations, including dental findings, periodontal screening, and assessment of the prosthodontic situation. OHRQoL was measured using the Oral Health Impact Profile (OHIP‐G14)	MNA and BMI was calculated		A strong relationship between nutrition and OHRQoL is found. Missing teeth were the strongest influential factor for malnutrition. Dementia, and decayed teeth were influential factors for MNA	Dental care should be encouraged in care homes to prevent malnutrition
23	Silva 2021	Impact of oral health on nutritional status, self‐perception of oral health and quality of life of institutionalised elderly	Care homes in Brazil	Cross‐sectional study	193 institutionalised older adults living in Brazil. F = 72%, *n* = 139) 80 years and above	To investigate the influence of oral health on nutritional status, self‐perception of oral health and HRQoL of institutionalised older adults	Geriatric Oral Health Assessment Index (GOHAI) evaluated self‐perception in oral health	MNA‐SF	The SF‐12 instrument measured HRQL	The oral health status has a limited impact on the nutritional status and does not impact the self‐perception of OHRQoL of institutionalised older adults	Qualitative studies should be designed to better understand the psychological aspects involved in the quality of life of institutionalised older adults
24	Wanderley 2021	Masticatory Function and Nutritional Status in Brazilian Institutionalised Elders: Influence of Denture Use	Care homes in Brazil	Cross‐sectional study	55 older adults from 7 long‐stay institutions in Brazil. Age 60 to 99 years old. (77.12%) F and (22.88%) M	To evaluate the influence of tooth loss and the use of removable dentures on chewing function and nutritional status of institutionalised older adults	Oral examination for assessment of masticatory function and teeth and denture use. The swallowing threshold determined by counting chewing cycles until swallowing	MNA‐SF		Toothless individuals without dentures had a lower swallowing threshold while partial toothless with dentures had better masticatory function	Regular monitoring of nutritional status by nutritionists, implement oral rehabilitation programs to maintain remaining teeth and denture replacement
25	Kuei‐Ru Chou 2023	A comprehensive assessment of oral health, swallowing difficulty, and nutritional status in older nursing home residents	Care homes in Taiwan	Cross‐sectional status	39 participants entered the study. The average was 80.4 ± 11.7 years	To highlight the importance of a comprehensive assessment of oral health, swallowing function, and nutritional status in long‐term care residents	Oral Health Assessment Tool (OHAT)), swallowing function (Functional Oral Intake Scale (FOIS)	Eating Assessment Tool (EAT)‐10), and (Mini Nutritional Assessment‐Short Form (MNA‐SF)		Oral consumption of a modified diet and swallowing problems and the nutritional status seemed to be related. Routine evaluation is necessary to preventing older adults' malnutrition	longitudinal, or interventional studies with larger sample size are required to determine relationships among oral health, swallowing function, and the nutritional status in older adults in care homes
26	Parisa Malekpour 2023	Investigating the perspectives of older adults in residential aged care on oral health‐related quality of life	Care home in Australia	Mixed‐method study	15 older adults from a not‐for‐profit residential aged care facility (RACF) in Australia. Age ranged from 68–95 years, with a mean age of 78.2 ± 8.3 years	To explore how older adults in care homes perceived that their oral health affected food preferences, attitudes towards food, their social interactions and their self‐esteem	The GOHAI questionnaire; used to assess links between participants' oral health status and their QoL	Semi‐structured interview about food habits in care home and analysed using TA		Oral health has a major influence on the physical and psychological well‐being of residents impacting their self‐esteem and interactions	These findings could be used by caregivers and policymakers to enhance residents' oral health and therefore diets and QoL
27	Nagata 2023	Effects of oral function and depressive tendencies on nutritional status in older adults requiring support or low‐level care: An investigation using path analysis	Care homes in Japan	Cross‐sectional study	106 older adults from care homes and private geriatric nursing homes	To investigate the effects of oral function and depressive tendencies on the nutritional status of older adults	Oral diadochokinesis (ODK), tongue pressure, and repetitive saliva swallowing test (RSST)	MNA‐SF	Depressive tendencies were evaluated using the Geriatric Depression Scale (GDS‐15) Diet‐Related Quality of Life (DRQOL) assessed using a specialised scale	Nutritional status was most affected by tongue pressure, Depressive tendencies and DRQOL, with gender differences indicating higher malnutrition rates in females	The need for interventions focusing on maintaining tongue pressure, addressing depressive symptoms, and improving dietary satisfaction to enhance nutritional status of older adults in care homes
28	Medeiros 2024	Masticatory function and mortality among older adults living in long‐term care facilities in Brazil	Care homes in Brazil	Longitudinal study	295 older adults living in LTC in 17 Brazilian care homes	To evaluate the link between masticatory function and mortality in older adults in LTC following 4 years	The number of remaining teeth assessed. Eichner index used to measure masticatory function. The use of dentures recorded. GOHAI and OHIP used to measure OHRQoL	Serum Albumin and BMI. MNA used to assess dietary habits, frequency and types of food	Fried's frailty phenotype components used to measure frailty	Objective measurement showed a 52% higher risk of early death in participants with poor masticatory performance, assessed via chewing gum colorimetric analysis, compared to those with good performance. Self‐reported masticatory dysfunction also indicated a 48% higher risk of early death	Promoting oral health care by providing appropriate dietary modifications to enhance chewing ability
Category 2: studies addressing the impact of nutrition on oral health											
29	MacEntee 1993	Predictors of caries in old age	Canada and USA	Longitudinal study	205 subjects over 65 years with natural teeth: group 1 (67%) lived in 9 LTC with severe functional disabilities. Group 2 (32.9%) lived independently	To measure the incidence of dental caries for 1 year To identify factors associated with the risk of caries	Oral examination to explore DMFT, oral hygiene, saliva flow, and estimated the presence of *Streptococcus mutans* and Lactobacilli in saliva samples at baseline and 1‐year follow‐up	Interviews at the outset and after a year on consumption of sugar (using food frequency) and use of medications		Increments of caries were associated with high baseline prevalence of caries, plaque index, high number of Lactobacilli, frequent sugar intake and poor oral hygiene	More attention should be given to the control of sugar consumption in LTC facilities. Fluoride and chlorhexidine and sugar substitute can reduce caries activity
30	Chalmers 2005	Caries incidence and increments in Adelaide nursing home residents	Adelaide, Australia	Longitudinal study	154 baseline residents/legal guardians. 111 completed a dental inspection. 41 were dentate. The great majority were aged 75+ years	To quantify coronal and root caries incidents and increments in residents of nursing homes	Questionnaires question about oral hygiene practices and dental history and oral examination assessing tooth status, caries incidence, and oral hygiene	Questionnaire to nursing home staff, family members and residents. Questions about types of food		Residents with eating and nutritional problems developed high increments of new coronal caries	Future studies will require larger nursing home samples to adequately identify caries risk factors in institutionalised older adults
Category 3: Studies addressing the impact of cognitive functions on oral health and nutrition											
31	Nordenram 1996	Alzheimer's disease, oral function and nutritional status	Stockholm, Sweden	Cross‐sectional study Clinical examination, MMSE and Muscle mass measured	40 Alzheimer's patients in care homes, and 40 age and gender matching control group living independently aged 75 years and older	To study difference in nutritional, and dental status in institutionalised patients with Alzheimer's disease and in cognitively ill health older adults at home	Chewing ability examined using Eichner Index for occlusion detection	BMI, eating habits, and food consistency were recorded	The mid‐upper arm circumstances (MAC) measured. Total body fat measured by Triceps skinfold thickness (TSF) Cognitive functions measured using MMSE	Nutritional status is influenced by dental status but the ability to eat unaided is strongly correlated to cognitive status	
32	Stewart 2007	Dental Health and Cognitive Impairment in an English National Survey Population	England	Cross‐sectional study (secondary analysis of English National Surveys)	65 and older living in community and care homes	To investigate the association between dental, nutritional and cognitive health	Dental status collected by interview including the degree of edentulism and dental impairment	MNA‐SF, and BMI	Cognitive functions measured using Abbreviated Mental Test Score (AMTS)	Poor dentition is associated with cognitive impairment. Nutritional status in people with cognitive impairment is recognised to be at risk	Although dental health did not explain the link between cognitive impairment and low BMI in this sample, other potential nutritional consequences require further investigation
33	Sadamori 2008	The relationships between oral status, physical and mental health, nutritional status and diet type in elderly Japanese women with dementia	Care home in Okayama, Japan	Cross‐sectional study	94 older women with dementia (mean age 89.6 ± 5.6 years) from a nursing home in Japan	To suggest methods for maintaining an adequate nutritional status for older adults with dementia by evaluating the relationships between oral status, physical, mental health, and feeding conditions	Occlusion and denture use assessed by a single examiner	BMI and serum albumin level. Self‐feeding ability (during daily life) of subjects was objectively evaluated by nurses	Mental state and daily activities were assessed with the NM scale for mental states and N‐ADL for daily living activities. Cognitive status was evaluated using the revised HDS‐R	A suitable type of diet and assistance with feeding could maintain the nutritional status of older patients with dementia if they are still feeding themselves	Participation of a dentist as part of nutritional support team is an important factor in the treatment of older adults with dementia
34	Sumi 2009	Relationship between oral function and general condition among Japanese nursing home residents	Japan	Cross‐sectional study: Oral function, cognitive function, and nutritional status assessed	79 residents of a nursing home in Japan (54 F and 25 M, age range: 65–95 years, mean age: 82.2 ± 8.5)	To clarify the relationship between oral function and general condition among Japanese nursing home residents	Gargling function test and Water drinking test	Serum albumin level and BMI	General function was assessed using MMSE for cognitive function and ADL for physical abilities	Oral function may play an important role in maintaining general condition in dependent older adults	To prevent decreases in cognitive function, ADL and nutritional status in dependent older adults, oral function should be improved
35	Ziebolz 2017	Oral Health and nutritional status in nursing home residents‐results of an explorative cross‐sectional pilot study	4 nursing homes in Northern Germany	Cross‐sectional pilot study	87 residents participated (mean age: 84.1 years; female: 72%, from 4 nursing homes	To investigate potential links between oral health (dentate or edentulous), other amnestic factors (age, smoking, dementia), and the risk of malnutrition in older populations	Oral examination by a dentist for dental status (DMFT) and assessment of periodontal treatment needs (PSR/PSI)	MNA		In nursing home residents, dementia was a better predictor of malnutrition risk than being edentulous	Further research is needed to determine the potential involvement of oral health as a cofactor for malnutrition in dementia
36	Lindroos 2019	Burden of Oral Symptoms and Its Associations with Nutrition, Well‐Being, and Survival among Nursing Home Residents	Helsinki, Finland	Cross‐sectional study	3123 residents living in assisted facilities and nursing homes in Helsinki, Finland	To explore how oral problems, cluster and whether their burden is associated with nutritional status, GI symptoms, psychology, and mortality among institutionalised residents	Questionnaire questions on Chewing and swallowing problems, and dry mouth (yes/no questions)	MNA GI symptoms (constipation, diarrhoea, and vomiting) charted Feeding dependency, food consistency, and food consumption were also assessed	Cognitive and physical functioning evaluated using validated questions from the Clinical Dementia Rating scale	The burden of oral health problems was associated in a phased fashion with poor health and psychological well‐being, malnutrition, and mortality	Clinicians should routinely assess older institutionalised residents' oral health status to improve residents' well‐being
37	vandeRijt 2021	Oral function and its association with nutrition and quality of life in nursing home residents with and without dementia: A cross‐sectional study	4 nursing homes in the UK	Cross‐sectional study	111 Residents aged 65 and older. 84 had dementia and 27 were without dementia	To compare oral function, nutritional status and quality of life (QoL) between residents with and without dementia	Interviews to determine denture use, swallowing, chewing, and xerostomia and Oral examination to determine orofacial pain & FTUs in the 2 groups	MNA‐SF		Oral function and nutritional status of residents with dementia was poorer than those without	Routine dental care in nursing homes is important to maintain sufficient chewing ability and treat pain causing oral health problems. Future research is needed to determine other possible risk factors for malnutrition
38	Suma 2022	Number of teeth, denture wearing and cognitive function in relation to nutritional status in residents of nursing homes	Nursing homes in Fukuoka City, Japan	Cross‐sectional study	162 participants in long‐term care (mean age: 87.7 ± 7.5 years, 26 M and 136 F) were admitted to 8 nursing homes in Fukuoka city, September 2013–March 2014	To test for the hypothesis: the link between the number of teeth and denture wearing with cognitive function is mediated by nutritional status in residents of care homes	Oral examination by a dentist evaluated number of teeth, denture use, and swallowing function (assessed with a stethoscope)	MNA‐SF	Cognitive function was assessed using the Clinical Dementia Rating (CDR)	There is an association between dental status and cognitive impairment in olders, and dentures and poor nutrition are involved in this association	Future research is required to gather detailed information regarding the usage of dentures with cognitive impairment in older adults
Category 4: Studies addressing the impact of functional abilities on oral health and nutrition											
39	Wardh 2004	Oral bacteria and clinical variables in dependent individuals at a special facility	Sweden	Cross‐sectional study	33 subjects from a special facility	To describe residents' oral flora in relation to other health variables and to classify the residents on different risk levels	Microbiological samples collected from dentate individuals and the dorsum of the tongue from non‐dentate individuals. The samples were microbially analysed to assess caries risk and mucosal infection	A 3‐day food records were kept by the caregivers		There was a link between high level of acid‐producing bacteria, functional impairment, and nutritional problems	The correlation between functional impairment and natural teeth is important to consider
40	Poulsen 2006	Nutritional status and associated factors on geriatric admission	Geriatric clinic in Copenhagen University Hospital	Cross‐sectional study	*n* = 196, mean age 83.7 years (69% F and 31% M)	To identify risk factors for undernutrition in geriatric patients on admission	Oral examination of mucosa, tongue, teeth, and denture status on admission	Nutritional risk factors, including poor appetite, oral cavity problems, constipation, and nausea or vomiting, were examined upon admission		Undernutrition is associated with oral health problems, age, physical abilities and appetite	It is recommended to routinely examine patients' mouth and physical and living condition on admission to improve care and prevent undernutrition
41	Kutsal 2014	Determination of the Relationships Between Anthropometric Characteristics and Level of Daily Activities, Nutritional Habits and Mouth‐Teeth Findings of the Elderly	Care homes in Turkey	Cross‐sectional study	84 residents aged ≥ 65 years. Mean age 80.5 ± 6.4 years	To determine the relationship between anthropometric characteristics and level of daily self‐care activities, nutritional habits and oral health status findings of the older adults	Interview: questions about oral hygiene and dentist visits and oral examination was performed	MNA and BMI	Obesity risk and muscle strength evaluated, and ADL used to assess dependency	Loss of teeth and reduced masticatory ability have a negative impact on nutritional status. The correlation between the muscle strength and ADL was statistically significant	Comprehensive Geriatric Assessment should be multidimensional and interdisciplinary. Diagnostic techniques should aim to evaluate not just medical but also functional skills in older adults
42	Keller 2017	Prevalence and Determinants of Poor Food Intake of Residents Living in Long‐Term Care	Canada	Cross‐sectional study	639 residents in 32 nursing homes in Canada, 628 with complete data. Average age was 86.3 ± 7.8 years and 69% were female	To measure energy and protein intake of long‐term care home LTCH residents	Oral exam by hygienists using a standardised oral health exam Dysphagia risk identified Coughing or choking during meal observations collected	3 non‐consecutive days of weighed food intake collected. Nutrient analysis conducted using Food Processor Nutrition Analysis Software	Data on medication, supplements, diet prescriptions, weight history, modified food texture diets, cognitive performance, depression, pain, and activities of daily living collected from health records, staff interviews, and standardised exams	There are many factors that could affect food intake in LTC: pureed food, restorative dining, eating assistance, and person‐centred care practices	Interventions focusing on pureed food, restorative dining, eating assistance, and person‐cantered care practices may support improved food intake and should be the focus of future study
43	deOliveira 2021	Health‐related quality of life of institutionalised older adults: Influence of physical, nutritional and self‐perceived health status	Brazil	Cross‐sectional study	The study participants were mostly female (*n* = 206, 59.9%) out of 344 with a mean age of 77.7 ± 9.1 years	To evaluate the influence of physical state, nutritional status and self‐perceived general and oral health on the QoL of institutionalised older adults in 2 Brazilian cities	Self‐perceived oral health status assessed using numerical scales. HRQoL measured using the 12‐Item Short Form Survey (SF‐12)	MNA and Body measurements	ADLs evaluated using the Katz scale, frailty assessed using a validated self‐perception instrument, and mental health assessed using the MMSE	A better physical state, nutritional status and self‐perceived general health leads to a better HRQoL in institutionalised older adults	HFA should promote programs that increase the independence and reduce the frailty of older adults residents in order to improve HRQoL
44	Julkunen 2021	Oral disease burden of dentate older adults living in long‐term care facilities: FINORAL study	Finland	Cross‐sectional observational study	Older adults living in LTC 65 years or older (*n* = 209). mean age 82 years. 68% suffered from dementia	To examine the association of oral disease burden (ODB) with health and functioning among dentate residents and to identify residents' poor oral health	Oral examination by two qualified dentists included inspection of lips, oral mucosa, dentures, oral wetness, speech, and eating	MNA and BMI The nurses also clarified residents' food consistency		Residents with low functioning had poor oral health, and ODB accumulated	Intervention research including care home staff and the implementation of the signs score‐6 items should be carried out
45	Irigoyen‐Camacho 2024	Relationship of Frailty, Nutritional Status and Oral Health‐Related Quality of Life in Mexico City Nursing Home Residents	Care homes in Mexico	Cross‐sectional study	286 older adults 65 years and over in care homes in Mexico City	To identify the link between nutritional status, OHRQoL and frailty	GOHAI used to measure OHRQoL	MNA	Fried component used to measure frailty	Improving oral health and nutritional status in older adults residing in nursing homes could play a significant role in reducing frailty and enhancing their overall quality of life	Involvement of geriatric physicians with dietitians, dentists, and dental hygienists to provide a multidisciplinary approach Improvements in the regulation of care homes and installing comprehensive programs for older adults
Category 5: Studies addressing the impact of care home staff on the oral health and nutrition											
46	Kayser‐Jones 1996	Mealtime in nursing homes: the importance of individualised care	USA	Longitudinal study	100 residents in two care homes with eating problems	To discuss the importance of providing an individualised care at mealtime	Oral examination to detect dysphagia by a dentist and speech by a researcher	Interviews with physicians, care home staff, residents, and their families about food. Residents who were not eating well were followed for 6 months until resolution of the problem or death		Three causes of malnutrition were reported; Lack of ethnic food, unrecognised dysphagia, and lack of assistance during mealtime	Care home staff can be trained to do brief oral assessment. Adequate number of staff is required to assist residents during mealtime “individualised care”
47	Kayser‐Jones 2002	Malnutrition, dehydration, and starvation in the midst of plenty: the political impact of qualitative inquiry	United States and Scotland	Qualitative observational study	LT residents of care home in US and in Scotland (*n* = 58), 50 nurses, 36 physicians and 50 family members	To investigate factors influencing nutritional intake of older adults in care homes	Oral health assessed via semi‐structured interview questions	Dietary analysis of 40 of 100 residents, BMI calculated, and fluid intake measured		Family members and attending physicians identified factors affecting nutritional intake as lack of individualised food, dysphagia, and insufficient staffing	Care for older adults in care homes needs to consider the problem of inadequate staffing especially for dietary assistance
48	Monaghan 2010	Oral health policy and access to dentistry in care homes	Wales	Cross‐sectional study	Care home managers in Wales	To explore factors which may facilitate or impede access to dental care	Questionnaire items about presence of natural teeth, denture use and dentists' visits	Questionnaire questions about lunch menus (for dentate) and feeding policy if residents have no natural teeth		Care home managers reported issues related to access to dental care, training needs for care home staff and assumptions that affect diet offered to residents. Only 50% of staff received oral hygiene training	Regular dental care should be offered to care home residents. Staff should be trained in oral hygiene provision. Community dental services should support homes in developing appropriate referral policies and provide skills to dentists
49	Jämsä 2024	Implementation of oral hygiene practices in nursing homes – a qualitative approach among supervisor nurses	Care homes in Finland	Qualitative study	19 supervisor nurses from care units in the six largest cities in Finland	To explore the implementation of oral care for residents in Finnish private care homes	Face to face interview questions on daily oral care practice	Face to face interview questions on snacks provided and mealtime		The need for staff better education, training, sugary snacks management, and improving the dental care services to enhance residents' oral health	

## 3 Results

### 3.1 Screening and Study Selection

**FIGURE 1:**
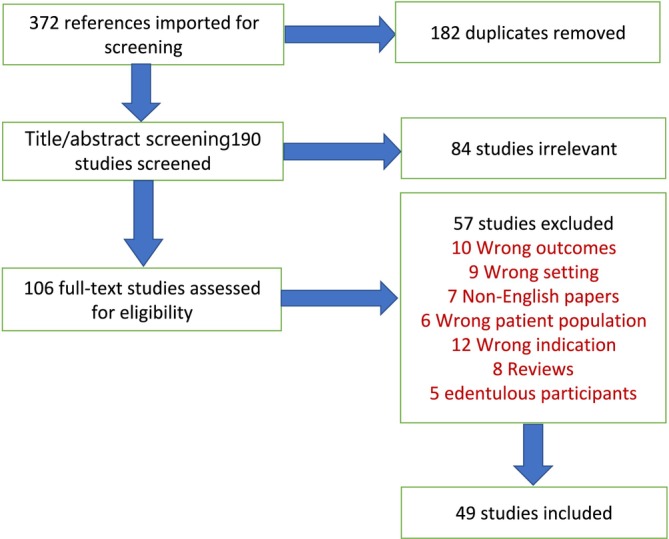
PRISMA flow diagram. [Colour figure can be viewed at wileyonlinelibrary.com]

A total of 372 articles were imported for screening. Following the removal of 182 duplicates, the title and abstract of 190 articles were checked against the eligibility criteria. As a result, 84 articles were rejected based on their title and abstract, leaving 106 articles to be evaluated for full‐text eligibility. At full‐text screening, 57 studies were excluded for the following reasons: 10 articles did not include data on diet/nutritional status and oral health outcomes, 9 were not conducted in care home settings, 7 articles were non‐English, 6 did not study older populations, 6 focused only on nutrition or oral health, 8 were reviews, and 5 were conducted on edentulous older adults (see PRISMA flow chart, Figure 1). Following screening, 49 eligible articles investigating the links between nutrition and oral health among dentate older adults in care homes published between 1989 and 2024 were included in the review.

The majority of included studies were cross‐sectional and aimed to determine an association/correlation between oral health and nutrition (*n* = 43) [19, 20]. Two interventional studies were included that tested approaches for improving oral health by regular professional tooth brushing and denture repair and studied the subsequent impact on nutrition [21, 22]. Four longitudinal studies were included: three investigated the relationship between caries, poor oral health, and sugar intake [23, 24, 25], while one assessed the impact of poor mastication on mortality due to its negative effect on nutrition [26]. The relevant data for each individual source of evidence is included in Table 1.

The studies included were conducted across various countries, with Japan (11 studies) having the highest number, followed by the USA [6], Germany, Brazil, and Finland (5 each). Canada and the UK contributed 3 studies each, while France, Taiwan, Australia, and Sweden had 2 studies each. Mexico, China, and Indonesia each contributed 1 study to the review.

### 3.2 Measures of Oral Health and Nutrition

The methods employed to measure the oral health and nutrition of care home residents in the reviewed studies exhibited some heterogeneity. The oral health of care home residents was assessed using clinical oral examinations in 28 out of the 45 studies [19, 20, 22, 23, 25, 27, 28, 29, 30, 31, 32, 33, 34, 35, 36, 37, 38, 39, 40, 41, 42, 43, 44, 45, 46, 47, 48, 49]. These examinations covered a range of factors including, dental status, denture usage, occlusal support, plaque index, tongue contamination, oral symptoms, periodontal health, and oral hygiene practices. The Oral Health Assessment Tool (OHAT) was utilised in one study to evaluate the health of lips, tongue, gums, saliva, teeth, dentures, oral hygiene, and recording dental pain [47]. Questionnaires and interviews were employed in 14 studies to gather insights into oral hygiene practices, chewing and swallowing difficulties, xerostomia, denture issues, and orofacial pain [24, 28, 30, 44, 50, 51, 52, 53, 54, 55, 56, 57, 58, 59]. Functional tests assessing masticatory function, gargling ability, and water drinking proficiency (swallowing function tests), the functional oral intake scale (FOIS), and tongue pressure were utilised in 6 studies [26, 36, 47, 60, 61, 62]. Microbiological sampling, utilised to evaluate caries risk and mucosal infection, was rare, appearing in only 1 study [63]. Some studies used a combination of methods to measure the oral health of care home residents.

Oral Health‐related Quality of Life (OHRQoL) was measured in 7 studies using the General Oral Health Assessment Index (GOHAI) and the Oral Health Impact Profile (OHIP), which assess functional and psychosocial effects of dental issues [21, 26, 39, 64, 65, 66, 67].

For nutritional assessment, the Mini Nutritional Assessment (MNA) and its short form (MNA‐SF) emerged as the most prevalent method, recorded in 25 studies [21, 22, 26, 32, 33, 34, 37, 38, 39, 41, 43, 44, 45, 46, 47, 49, 51, 53, 54, 55, 56, 62, 65, 66, 67]. Dietary surveys, records of food consistency, including the Eating Assessment Tool (EAT‐10), a questionnaire allowing adults to self‐assess their swallowing difficulties, were conducted in 14 studies [19, 20, 24, 27, 28, 30, 31, 35, 36, 48, 57, 58, 60, 63]. BMI measurement, utilised in 14 studies, served as an indicator of participants' nutritional status based on their body mass index [26, 29, 34, 39, 40, 42, 46, 49, 50, 52, 53, 55, 57, 61]. Serum albumin levels were assessed in 5 studies as a marker of malnutrition [21, 26, 29, 42, 61]. Additionally, structured and semi‐structured interviews were employed in 4 studies to elucidate participants' food habits, dietary aspects, and fluid intake [23, 25, 59, 64]. Some studies used combined methods to measure the dietary intake and nutritional status of care home residents.

### 3.3 Narrative Synthesis of the Findings

**FIGURE 2:**
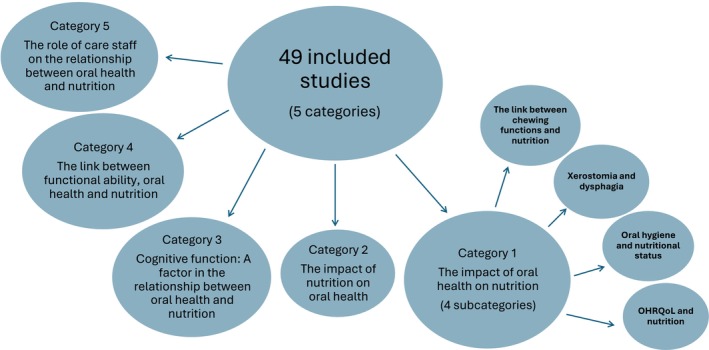
A diagram showing categorisation of included studies. [Colour figure can be viewed at wileyonlinelibrary.com]

The studies were categorised based on *their aims*. The largest group (*n* = 28) examined how reduced oral function due to chewing problems (caused by tooth loss and insufficient tongue strength), poor oral hygiene, xerostomia, or dysphagia might be linked to nutritional deficiencies in older adults (Category 1). A smaller group (*n* = 2) explored the impact of dietary habits such as high sugar intake on oral health (Category 2). Other studies (*n* = 19) looked at factors such as cognitive function (Category 3), functional abilities (Category 4), and staff performance in care homes (Category 5) and their potential influence on oral health and nutrition. Many studies fell into multiple categories due to the interrelated nature of these factors (see Figure 2 below). Categories and subcategories are shown in Table 1.

Table 1 below presents the extracted data from all eligible studies, organised by category and chronologically within each category. Studies addressing multiple aims are included in the primary aim's category. Papers in Category 1 are numbered 1–28, Category 2 are 29–30, Category 3 are 31–38, Category 4 are 39–45, and Category 5 are 46–49. The narrative synthesis includes the author's name and corresponding number from Table 1.

#### 3.3.1 The Impact of Oral Health on Nutrition

The largest category of the studies in this review investigated the impact of oral health on nutrition (*n* = 28) [19, 20, 21, 22, 26, 27, 28, 29, 30, 31, 33, 34, 35, 36, 37, 38, 39, 41, 47, 50, 51, 52, 60, 62, 64, 65, 66, 68]. Based on the aspect of oral health under investigation, the studies were classified under four subcategories as follows.

##### 3.3.1.1 The Link Between Chewing Functions and Nutrition

The 18 studies in this subcategory identified a connection between decline or loss of chewing and swallowing functions and nutritional problems in older adults in care homes [19, 20, 21, 26, 27, 30, 31, 33, 34, 36, 37, 38, 41, 50, 51, 60, 62, 66]. These studies suggested that poor chewing function may contribute to decreased appetite and poor nutrition, potentially exacerbating malnutrition and frailty. However, as these are cross‐sectional studies, the direction of this relationship remains unclear.

Decline in chewing ability can be caused by loss of natural teeth, or the loss of tooth contact pairs [19, 20, 26, 27, 30, 31, 33, 34, 36, 41, 50, 51, 66]. The lack of teeth or dentures impairs chewing ability, requiring a change in food consistency (from regular food to soft or pureed), which in turn negatively affects nutritional status. Authors recommended maintaining natural teeth and tooth occlusion and denture provision/repair to improve chewing [19, 20, 26, 27, 30, 31, 33, 34, 36, 41, 50, 51, 66]. Moreover, Saarela et al. and Medeiros et al. linked being edentulous without a prosthesis and chewing difficulties to a higher risk of mortality due to poorer nutritional status in care home residents. However, authors recommended further research to validate the predictive value of this risk, emphasising the need for cautious interpretation within a life‐course framework while considering methodological challenges [26, 51].

Kuijk et al. also investigated the link between missing teeth and malnutrition but found no direct connection. However, they did suggest an association between malnutrition, untreated dental caries, diminished cognitive functions, and increased dependency among older adults in a bidirectional manner (see also category 3 and 4) [38]. However, the use of a cross‐sectional study design did not provide any information on potential time lags between tooth loss and the onset of malnutrition.

The second reported cause for decline of chewing and swallowing functions is diminished tongue strength/pressure, as reported by Izumi et al., Nakagawa et al., and Nagata et al. [37, 60, 62]. They suggested that participants with inadequate tongue strength, often resulting from frailty and a lack of molar occlusion due to tooth loss, chewed less and ingested large‐sized particles. This not only increased the risk of choking or aspiration but also compromised digestion and nutrient absorption, which in turn can contribute to poor nutritional intake and overall health decline in care home residents [60].

##### 3.3.1.2 Xerostomia and Dysphagia

Four studies investigated the link between xerostomia (dry mouth) or dysphagia (swallowing difficulty) and older adults' nutritional status [25, 28, 47, 50]. Loesche et al. investigated the relationship between xerostomia and food avoidance through clinical examinations and surveys, finding that older adults with xerostomia were 14 times more likely to report issues with their dentures. Many of these individuals were on xerogenic medications, and the authors noted a significant correlation between xerostomia and food avoidance, leading to dietary restrictions and potential undernutrition [28].

Wang et al. studied chewing and swallowing problems (PCS) and found significant associations between PCS, malnutrition, cognitive performance, and social engagement, suggesting that staff education on monitoring PCS is crucial [50]. Chou et al. linked dysphagia to malnutrition risks using assessment tools OHAT, FOIS, and EAT‐10, revealing significant associations between poor oral health, impaired swallowing function, and increased malnutrition risk. The study emphasised the need for regular swallowing assessments to prevent malnutrition [47].

Kayser‐Jones et al. conducted an observational study highlighting the importance of personalised care during mealtime for residents with dysphagia. They found that dysphagia prevalence caused mealtime challenges and malnutrition through food avoidance. Furthermore, staff shortages meant personalised care was not always feasible [25].

##### 3.3.1.3 Oral Hygiene and Nutritional Status

Barbe et al. examined the relationship between oral hygiene and the nutritional status of older adults using an interventional study evaluating professional tooth brushing in care homes. Residents received oral hygiene examinations followed by professional tooth brushing every three weeks for three months, improving both oral hygiene and nutritional status, especially in residents without dementia. The study suggested even better results might be achieved with more frequent cleanings [22].

Additionally, Wardh et al.'s pilot study analysed oral microorganisms in plaque, finding that high levels of acid‐producing bacteria correlated with poor nutritional and functional health. This emphasised the need to improve oral hygiene to enhance nutritional status [63]. The study was categorised in category 4 based on the primary aim (see also category 4).

Moreover, Monaghan et al. surveyed care home managers about dental care access and residents' ability to chew, revealing a lack of staff training in providing oral hygiene support. This study highlighted the role of care home staff in promoting oral health and nutrition among older adults [58]. This study has been included in category 5 since it contributes to understanding the role of care home staff in promoting oral health and nutrition among older adults residing in care homes (see also category 5).

##### 3.3.1.4 Oral Health‐Related Quality of Life OHRQoL and Nutrition

Five studies investigated the relationship between Oral Health‐Related Quality of Life (OHRQoL) and nutrition in older adults in care homes employing various methodologies [21, 39, 64, 65, 66].

Malekpour et al. used the GOHAI questionnaire, semi‐structured interviews, and thematic analysis to study the perceptions of care home residents about how their oral health affects their dietary choices, social engagement, and self‐esteem. The study found that poor oral health led to physical discomfort, difficulty chewing, and dysphagia, impacting dietary choices and potentially leading to malnutrition and social isolation [64]. Issues such as tooth loss, denture‐related difficulties, and swallowing problems required residents to adapt their eating habits by avoiding certain textures or relying on modified foods. Some participants struggled with chewing foods like meat and needed softer preparations, while others with severe swallowing difficulties were restricted to pureed diets [64].

Schmalz et al. examined the impact of oral health on nutritional status and OHRQoL in a German care home using health records, MNA, OHIP‐G14 and oral examinations. They found that missing teeth and poor oral health were significant predictors of malnutrition, though the effect on OHRQoL was relatively small. The study highlights that poor oral health negatively affects nutritional status. However, the study did not report detailed effect sizes, making the strength of these associations unclear. Further studies with clearer adjustment for confounders and reported effect sizes are needed to better understand these relationships [39].

Silva et al. used the GOHAI questionnaire to measure OHRQoL and found that while poor oral health conditions were prevalent, residents' self‐perception of oral health was generally positive. The study suggested that factors beyond oral health, such as social, cultural, and psychological influences, affect how older adults perceive their oral health status [65].

Wostmann et al. conducted an interventional study to assess the impact of denture provision on nutritional status and OHRQoL. Using the OHIP, masticatory function tests, and MNA, they found that while masticatory efficiency improved after prosthetic treatment, there were no significant improvements in nutritional status or OHRQoL. The study recommended considering dietary consultation alongside prosthetic treatment [21]. This study was also included in the first subcategory (chewing function and nutrition).

#### 3.3.2 The Impact of Dietary Intake on Oral Health

Two longitudinal studies identified in this review investigated the impact of sugar intake on oral health for care home residents [23, 24].

MacEntee et al. conducted a survey and dental examination of care home residents to identify factors associated with dental caries. They assessed sugar intake, oral hygiene, salivary flow, and oral bacterial count, particularly Lactobacilli, at baseline and one year later. After one year, the study found that high Lactobacilli levels caused by frequent sugar intake were linked to poor oral hygiene and dental caries in older adults. They suggested that regulating sugar consumption in long‐term care (LTC) facilities, along with using fluoride, chlorhexidine, and sugar substitutes, could help reduce caries. They also called for more research on the effects of excessive sugar consumption on the oral and general health of institutionalised older adults [23].

Chalmers et al. evaluated dental caries in older adults residing in Adelaide nursing homes over a year. Using questionnaires, interviews, the Katz Index for functional status, and the MMSE for mental state, they gathered data on socio‐demographics, oral hygiene practices, and medical history. Dentists assessed coronal and root caries. They found a high incidence of new coronal (64.4%) and root caries (48.5%) with greater increments over one year compared to healthier, community‐dwelling older adults over 3–5 years. Residents who had lost weight or had restricted diets had significantly higher coronal caries increments. The study highlighted the correlation between declining nutritional status and increased dental caries, emphasising the need to consider residents' nutritional status in dental care planning in care homes [24].

#### 3.3.3 Cognitive Function: A Factor in the Relationship Between Oral Health and Nutrition

Eight studies explored the relationships between cognitive health, oral health, and nutrition among older adults [40, 43, 45, 53, 54, 61, 69, 70]. These studies hypothesised that cognitive health might play a role in influencing nutrition and oral health outcomes, potentially acting as an effect modifier that exacerbates the impact of oral health issues on nutrition, or as part of a broader causal pathway where poor cognitive health can contribute to malnutrition and oral health decline. However, the cross‐sectional nature of many of these studies limits the ability to confirm specific causal pathways.

The Mini‐Mental State Examination (MMSE) was utilised to assess cognitive health alongside evaluations of nutritional and oral health. In these, malnutrition was associated with cognitive impairments such as dementia, highlighting cognitive health as an additional factor alongside conditions like tooth loss, which was more prominently linked to malnutrition in studies from subcategory 1 [40, 54, 56, 61, 70].

van de Rijt et al. found that residents with severe cognitive impairment were twice as likely to be malnourished or at risk of malnutrition compared to those with normal cognitive function. The risk increased for residents with chewing problems due to dentures [69]. In an observational study, Ziebolz et al. found that dementia was a better predictor of malnutrition risk than being edentulous, suggesting that malnutrition could exacerbate cognitive decline [43]. Adding to the link between cognitive functions and nutrition, Suma et al. found that care home residents with 0–19 natural teeth without dentures had a higher risk of having significant cognitive impairment than those with 20 or more teeth [45]. In the same context, Sumi et al. suggested that to prevent deteriorating cognitive functions in dependent older adults, nutritional status and oral functions should be improved [61]. Therefore, it was recommended that routine dental care and nutritional support during mealtime be implemented in older adults with dementia to maintain a satisfactory intake of calories and to avoid deteriorating cognitive functions [54, 70].

These findings suggest that cognitive impairment may exacerbate the nutritional and oral health challenges faced by older adults, though further research is needed to define its precise role within these complex relationships.

#### 3.3.4 Functional Ability, Oral Health and Nutrition

Seven cross‐sectional studies in this review examined the relationship between functional ability, oral health, and nutrition in care home residents. Functional ability refers to an individual's capacity to perform essential daily activities such as eating and walking, and as it declines with age, it often leads to frailty [46, 47, 48, 49, 55, 56, 63, 67].

de Oliveira et al. used the Katz scale to evaluate daily life activities (ADL), while Irigoyen‐Camacho et al. used the Fried scale to assess frailty. Their findings indicated that better physical status correlates with improved nutritional status and oral health. Moreover, improving oral health and nutritional status reduces frailty and enhances overall quality of life [56, 67]. However, since these studies are cross‐sectional, a longitudinal study is needed to establish a clearer understanding of the causal relationship.

Kutsal et al. studied the relationship between muscle strength, ADL, nutritional habits, and oral health, finding that reduced ADL is linked to muscle mass loss, malnutrition, and tooth loss [55]. Furthermore, Keller et al. identified mealtime difficulties, noting that as residents' functional abilities decline, they require more assistance to avoid choking, particularly those with dysphagia. They emphasised that carers must be aware of the increasing need for assistance with daily activities to prevent malnutrition due to oral health issues [48]. Keller et al. also found that dependency during eating is associated with higher morbidity (especially aspiration pneumonia) and mortality. Poor gargling function was linked to difficulty feeding oneself and increased risk of aspiration pneumonia. Therefore, maintaining proper gargling function is crucial to prevent inhaling oral pathogens [48]. It is therefore recommended by Poulsen et al. to routinely examine a resident's mouth and physical abilities on admission to care homes in order to improve care and prevent undernutrition [46]. Also, programs that increase the independence and reduce the frailty of older adult residents should be promoted in care homes to improve HRQoL, as suggested by deOliveira et al. [56]. Kuijk et al. suggested studying the length of stay in care homes as an important characteristic that may have an impact on the residents' functional ability and malnutrition [38].

#### 3.3.5 The Role of Care Staff on the Relationship Between Oral Health and Nutrition

Four studies in this review addressed the topic of staff roles in care homes with the objective of enhancing residents' nutritional status and oral health [25, 57, 58, 59].

Monaghan et al. interviewed care home managers and found that many carers are unaware of the importance of maintaining residents' oral health. They emphasised the need for regular dental care, staff training, and collaboration with dental professionals to improve residents' oral health and nutritional status [58]. Similarly, Jämsä et al. interviewed supervisor nurses in Finnish private care homes and found that daily sugary snacks (see also category 2) and inadequate staff education on oral health contribute to poor resident oral health. They recommended better staff training, managing sugary snacks, and using effective care aids like electric toothbrushes (See also subcategory 3), enhancing onsite dental services, and fostering co‐operation among healthcare professionals [59].

Moreover, Sioni et al. conducted a survey and clinical oral examination to explore the connection between oral health and nutrition in long‐term care (LTC). They found that staff assistance with oral health routine for frail older adults was linked to improvements in both oral health and nutritional status [68].

Kayser‐Jones et al. conducted a qualitative observational study on the importance of individualised care during mealtimes. They identified factors contributing to malnutrition, dehydration, and weight loss, including lack of personalised care, inattention to dysphagia and oral health, insufficient staffing, and lack of professional supervision [25]. After presenting some tragic details about the level of care in care homes, the author concluded, “Can a society call itself civilized if it does not provide sensitive, humane care to older people during the last days of their lives?” Kayser‐Jones 1996.

## 4 Discussion

### 4.1 Summary of Evidence

The primary aim of this scoping review was to identify, evaluate, and synthesise all relevant research data about the relation between oral health and nutrition in older adults living in care homes. 49 studies were identified, spanning from 1989 to 2024. Most of the eligible studies used a cross‐sectional design and highlighted a negative relationship between oral health issues, such as chewing difficulties, xerostomia, and dysphagia, and reduced dietary intake and nutritional deficiencies [19, 20, 21, 22, 23, 24, 26, 27, 28, 29, 30, 31, 33, 34, 36, 37, 38, 39, 40, 41, 45, 47, 50, 51, 52, 59, 60, 61, 62, 64, 66, 67, 68]. On the other hand, oral health interventions such as denture provision or repair and professional tooth brushing were associated with improved nutritional status, as measured by the Mini Nutritional Assessment (MNA) or Body Mass Index (BMI) [22, 66]. Xerostomia was a common issue linked to malnutrition, along with dysphagia and tooth loss without prosthetic replacement. Impaired chewing ability due to tooth loss and/or insufficient tongue strength could lead to a preference for a softer diet, potentially highlighting the importance of maintaining chewing ability for a varied diet and better nutritional status [36, 71].

Only two studies focused on the impact of dietary intake on oral health, both highlighting the need to minimise sugar intake to prevent caries and improve dentition and nutritional status [23, 24] (see category 2 section in the results).

Additional factors affecting oral health and nutrition included cognitive impairment caused by dementia and Alzheimer's disease, rising levels of resident dependence, particularly in oral care and during mealtimes, and staffing issues such as insufficient staff, training needs, and lack of individualised care [25, 40, 42, 43, 45, 46, 48, 49, 53, 54, 55, 56, 57, 58, 61, 63, 69].

### 4.2 Gaps in Literature

The secondary aim of this review was to identify gaps in the literature regarding the impact of dietary intake and nutritional status on the oral health of older adults in care homes. Malnutrition and many oral diseases are aging‐related issues with a bidirectional relationship that can lead to mortality. However, most research has focused on how oral health affects diet and nutrition, rather than the reverse [72].

Our review highlights a lack of recent studies (post‐2005) examining the effects of the frequency and/or timing of dietary intake, such as high sugar, on oral health in older adults. Few studies, like those by Chalmers et al. [24] and MacEntee [23], have investigated how poor nutrition can lead to oral health problems like caries. This gap may stem from the perception that oral health issues are less serious than malnutrition, despite the fact that high sugar consumption can contribute to both poor oral health and malnutrition.

In terms of study design, most studies in this review used cross‐sectional designs, with only two qualitative studies [25, 59] and four longitudinal studies included [22, 23, 24, 25]. More intervention studies are needed to explore care home residents' experiences and perceptions of sugar intake and its impact on oral health. Additionally, further longitudinal studies are required to assess the long‐term effects of sugar consumption on oral health outcomes in this population.

### 4.3 Authors Recommendations

Many authors of the eligible studies provided recommendations for future research and practice around oral health and nutrition in care homes, which are outlined in the results table (Table 1).

For improving care in care homes, several studies suggested training staff in providing individualised oral care and mealtime assistance to meet residents' specific needs [25, 50, 57, 58]. Caregivers' essential role in daily oral care routines and collaboration with dental professionals was also emphasised [32, 46, 50]. Regular dental examinations to identify issues early, preserve oral function, and training programmes to enhance staff skills and awareness about oral health were recommended [25, 33, 34, 51].

Some authors recommended integrating dentists and dental hygienists into the care homes healthcare team for better monitoring of oral health and denture maintenance, which enhances residents' masticatory function and reduces malnutrition‐related complications [29, 34, 39, 42, 44]. Caregivers were advised against altering meal consistency upon requests to preserve chewing function, and future research should define optimal food consistency in care homes [36, 71].

To address accessibility issues, the use of a mobile dental team was proposed, ensuring residents receive necessary dental services even when traditional visits are difficult [20]. A multidisciplinary approach involving dentists, nutritionists, and care home staff was suggested for comprehensive care [47, 66].

Finally, the need for further research, including longitudinal studies, was highlighted to explore the relationships among oral health, nutritional status, and overall well‐being in care home residents. This research can inform evidence‐based practices and interventions, ultimately improving the QoL for this vulnerable population [36, 52]. longitudinal studies can monitor and evaluate residents' functional skills. This could enhance residents' independence and reduce frailty that is found to be linked to poor oral health and malnutrition [21, 32, 37, 44, 52, 55].

Furthermore, qualitative methods have been recommended by the authors for developing a more comprehensive understanding of the psychological aspects that impact OHRQoL and overall QoL. That could look into residents' subjective perceptions and experiences, offering insight on the psychological factors influencing their oral health and general well‐being [65].

### 4.4 Implications of Findings for Care Homes

This scoping review finds that regular dental exams, proper oral hygiene, and early interventions can help care home residents maintain better oral health, including natural teeth retention. Improved oral health leads to fewer dental problems, better comfort, and enhanced nutritional status. By understanding the link between oral health and nutrition, care homes can proactively support residents' nutritional needs, such as assessing chewing ability, providing eating assistance, and making necessary dietary adjustments, ultimately leading to better overall health.

It is recommended to implement training programmes for care home staff, focusing on oral health care, individualised care during mealtime, and monitoring frailty among residents. Additionally, engaging with dental professionals, nutritionists, and other healthcare professionals allows care homes to access specialised expertise and resources to support residents' oral health and nutrition.

### 4.5 Strengths and Limitations

In this scoping review, an extensive search strategy was implemented to identify relevant studies focusing on nutritional status and oral health in care homes. The review process involved the active participation of two reviewers at each stage. To ensure methodological accuracy, a pre‐established protocol was developed, thoroughly reviewed, and adhered to throughout the review process. However, it is important to acknowledge certain limitations. One limitation is the possibility of excluding relevant studies due to the omission of some publications (non‐English and grey literature). Additionally, the absence of a quality assessment, although not necessary for a scoping review [17], is another limitation of this review as it may affect the overall quality and reliability of the evidence. Furthermore, some of the included studies used BMI and serum albumin as measures of nutritional status, which are considered flawed indicators of undernutrition in aged care. These measures may not accurately reflect the nutritional status of older adults, potentially affecting the findings and interpretations of the review.

## 5 Conclusions and Recommendations

This review examined 49 studies that investigated the relationship between oral health and nutrition among older adults who live in care homes. The findings suggest that poor oral health is associated with malnutrition in this age group. Additionally, the review highlights that oral health and nutrition in care homes can be influenced by cognitive health, functional abilities, and staffing. Residents with cognitive impairments, such as dementia, are at a higher risk of malnutrition and oral health issues due to difficulties in self‐care and making appropriate dietary choices. Functional limitations, like reduced muscle strength and the ability to perform daily activities, contribute to mealtime difficulties and dependency, which increase the risk of malnutrition and poor oral health. Staffing levels and training also play a crucial role, as care staff may lack awareness and skills in maintaining residents' oral hygiene, further compromising both oral health and nutritional status.

Future research should attempt to clarify the relationships between oral health and nutritional status, including causation and interactions. The quality of existing qualitative and longitudinal studies on this topic was found to be insufficient. Therefore, it is recommended that future research should address this gap by conducting more in‐depth studies, incorporating the suggestions provided by the authors of the papers included in this review.

## Author Contributions

All authors had meaningful contributions to the review. Aziza Sallam, Noleen K. McCorry and Gerry McKenna conceptualised and designed the study. Aziza Sallam and Kerry B.D. Campbell were responsible for data collection. Aziza Sallam and Noleen K. McCorry analysed and interpreted the data. Noleen K. McCorry and Gerry McKenna provided critical review and revisions as well as the remaining authors. All authors have read and approved the final version of the manuscript.

## Conflicts of Interest

The authors declare no conflicts of interest.

## Data Availability

The data supporting the findings of this study are available upon request from the corresponding author.
